# Synergistic PA and HA mutations confer mouse adaptation of a contemporary A/H3N2 influenza virus

**DOI:** 10.1038/s41598-019-51877-4

**Published:** 2019-11-12

**Authors:** Mariana Baz, Zeineb M’hamdi, Julie Carbonneau, Sophie Lavigne, Christian Couture, Yacine Abed, Guy Boivin

**Affiliations:** 1 Research Center in Infectious Diseases of the CHU of Québec and Laval University, Québec City, Québec, Canada; 2 Quebec Heart and Lung Institute, Department of Anatomopathology and Cytology, Laval University, Québec City, Québec, Canada

**Keywords:** Influenza virus, Infection

## Abstract

The mouse is the most widely used animal model for influenza virus research. However, the susceptibility of mice to seasonal influenza virus depends on the strain of mouse and on the strain of the influenza virus. Seasonal A/H3N2 influenza viruses do not replicate well in mice and therefore they need to be adapted to this animal model. In this study, we generated a mouse-adapted A/H3N2 virus (A/Switzerland/9715293/2013 [MA-H3N2]) by serial passaging in mouse lungs that exhibited greater virulence compared to the wild-type virus (P0-H3N2). Seven mutations were found in the genome of MA-H3N2: PA(K615E), NP(G384R), NA(G320E) and HA(N122D, N144E, N246K, and A304T). Using reverse genetics, two synergistically acting genes were found as determinants of the pathogenicity in mice. First, the HA substitutions were shown to enhanced viral replication *in vitro* and, second, the PA-K615E substitution increased polymerase activity, although did not alter virus replication *in vitro* or in mice. Notably, single mutations had only limited effects on virulence *in vitro*. In conclusion, a co-contribution of HA and PA mutations resulted in a lethal mouse model of seasonal A/H3N2 virus. Such adapted virus is an excellent tool for evaluation of novel drugs or vaccines and for study of influenza pathogenesis.

## Introduction

Type A and B influenza viruses cause substantial morbidity and mortality each year, imposing significant economic and health burden. Influenza viruses are enveloped negative-stranded RNA viruses with a segmented genome belonging to the *Orthomyxoviridae* family^1^. Influenza A viruses are composed of eight genetic segments encoding several different structural and non-structural/regulatory proteins^2^. The hemagglutinin (HA), neuraminidase (NA) and matrix 2 (M2) proteins are embedded in the viral envelope, whereas viral polymerase proteins (PB1, PB2, and PA), nucleoprotein (NP), matrix 1 (M1), non-structural protein 1 (NS1) and nuclear export protein (NEP) are localized inside the virion. Subtypes H1N1 and H3N2 of influenza A virus are currently circulating in the human population. Since the emergence of the H3N2 pandemic virus in 1968, influenza seasons in which A/H3N2 viruses are predominant over those of A/H1N1 strains have been associated with a greater number of hospitalizations and deaths.

The susceptibility of mice to influenza viruses depends on both mouse and viral strains. In general, mice are not naturally infected with seasonal influenza viruses and infections are typically asymptomatic with little or no viral replication. The basis of this phenomenon is that inbred mice possess an interferon-inducible restriction factor known as Mx1^3^. However, most influenza strains can be experimentally adapted for mouse virulence by serial lung-to-lung passages^4,5^. Mouse adaptation results in the acquisition of functions that are critical determinants of virulence with increased viral titers in the lungs and increased pathogenesis and mortality. Mouse-adapted influenza mutants usually induce pathologic changes in the bronchi or lungs, possess an increased ability to infect alveolar cells and can cause lethal pneumonitis^4–6^. The main advantage of using mice is that the pulmonary pathology is similar to that seen in the cases of viral pneumonia in humans^7^.

Several factors affecting influenza virus host range and virulence in mice have been identified. The influenza virus hemagglutinin (HA) is a primary determinant for mouse adaptation. Mutations in the HA receptor binding or protease cleavage sites as well as gain or loss of glycosylation sites alter virulence, replication, tissue tropism and host range^8–14^. The viral polymerase, the nucleoprotein (NP) and the viral RNA genome form the ribonucleoprotein (RNP) complex, which is required for both transcription and viral genome replication^15^. The influenza polymerase is a heterotrimeric protein containing three virally- encoded subunits: PB1, PB2 and PA and adaptive mutations in these proteins also contribute to overcome species’ barriers^16^. The majority of the mammalian adaptive substitutions occur in the PB2 protein; E627K and D710N are two well-characterized substitutions in PB2 protein, which are critical for mammalian adaptation in multiple subtypes of avian influenza viruses^17–22^. PB1 and PA have also been implicated in mouse lung virulence and play a critical role in mammalian adaptation^14,23–29^. Finally, the non-structural 1 (NS1) protein is important for blocking an innate cellular immune response against the virus and may be crucial for influenza virus pathogenicity^30,31^.

An old seasonal mouse-adapted A/H3N2 influenza virus such as A/Victoria/3/75 is frequently used for the evaluation of antivirals and vaccines. In order to use a contemporary seasonal A/H3N2 virus, we generated a mouse-adapted A/H3N2 influenza virus, A/Switzerland/9715293/2013, by performing sequential lung-to-lung blind passages in immunosuppressed (IS) mice followed by additional lung-to-lung blind passages in immunocompetent (IC) C57/BL/6 mice. We subsequently characterized the *in vitro* and *in vivo* replication kinetics, histopathology, cytokine/chemokine profiles of the adapted virus and we elucidated the molecular basis of pathogenicity using a reverse genetics system. Herein, we report on a novel combination of substitutions in HA (N122D, N144E, N246K and A304T) and PA (K615E) proteins responsible for the adaptation and increased pathogenicity of a contemporary A/H3N2 seasonal virus in mice.

## Results

### Mouse adaptation

**Figure 1:**
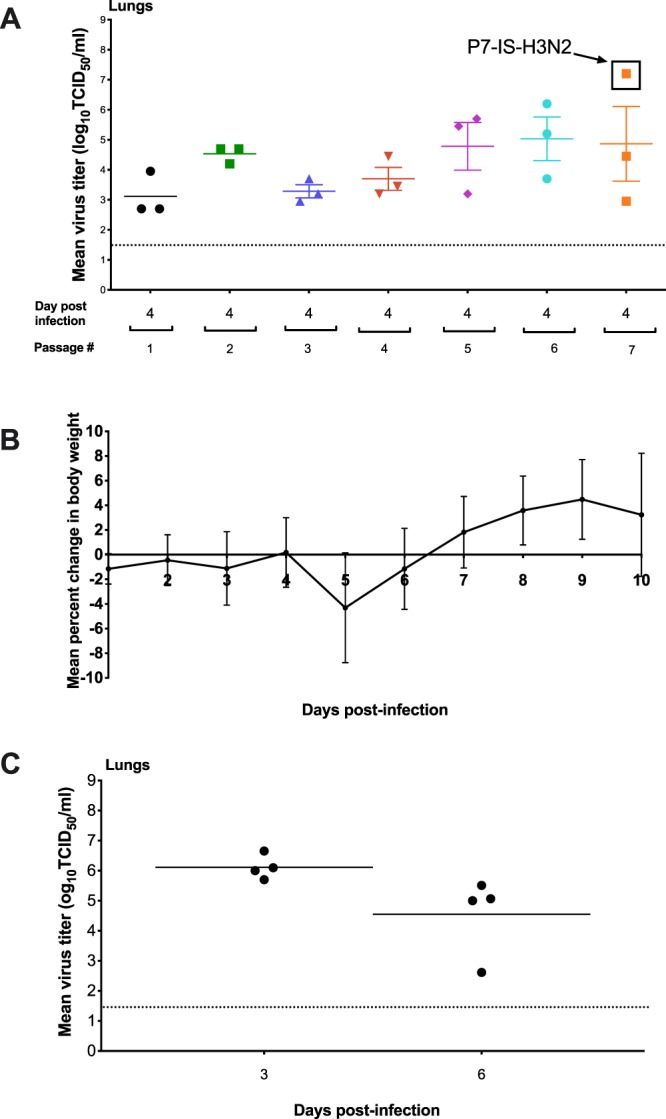
Mouse adaptation. (**A**) Lung-to-lung passages were done in pharmacologically-induced IS mice (n = 3) initially infected with 2.5 × 10^5^ PFU/50 µl of the parental H3N2 virus, P0-H3N2. Virus titers in the lungs from passages 1 to 7 are shown. (**B**) Mean percentage of weight loss of IC mice infected with an inoculum of 2.5 × 10^4^ PFU/50 µl of P7-IS-H3N2 virus (highlighted in figure A). Mice were observed daily for 10 days for clinical signs of illness, including weight loss, ruffled fur, and hunching. (**C**) Mean virus titers in the lungs of mice on days 3 and 6 post-infection. Titers are expressed in log_10_ TCID50/ml. The dashed horizontal line indicates the lower limit of detection.

Murine adaptation was carried out by lung-to-lung blind passages. First, IC C57BL/6 mice were infected with an inoculum of 2.5 × 10^5^ PFU/50 µl of an avirulent influenza strain for mice: A/Switzerland/9715293/2013 (P0-H3N2). Four serial blind passages were performed followed by titration of lung homogenates. No virus titer was detected in any of the four passages (data not shown). A pharmacologically-induced IS mouse model was then used to favor the replication of the parental virus^32–35^. A group of three mice were given a dose of cyclophosphamide (100 mg/kg) one day before intranasal infection with 2.5 × 10^5^ PFU/50 µl of the parental A/H3N2 virus (P0-H3N2). After seven consecutive blind passages, virus titers of lung homogenates were assayed for each passage (Fig. 1A). IS mice infected with the parental A/H3N2 virus generated virus titers ranging from 3.1 log_10_ TCID_50_/mL (passage 1) to 4.9 log_10_ TCID_50_/mL (passage 7). One mouse from the 7^th^ passage had a titer of 7.2 log_10_ TCID_50_/mL and was referred as the P7-IS-H3N2 virus. This strain was plaque purified and the virus was amplified in Madin-Darby canine kidney (MDCK) cells overexpressing the α2.6 sialic acid receptor (ST6-GalI-MDCK cells). P7-IS-H3N2 virus was then used to infect eight IC mice with an inoculum of 2.5 × 10^4^ PFU/50 µl. Although mice did not show significant weight loss (mean of 4.3%) on day 5 (Fig. 1B) and no mortality was observed, mean virus titers in the lungs on days 3 and 6 post-infection (p.i.) were 6.1 and 4.5 log_10_ TCID_50_/mL, respectively (Fig. 1C). Eight additional lung-to-lung passages in IC mice using the P7-IS-H3N2 virus were performed resulting in titers ranging from 6.5 log_10_ TCID_50_/mL (passage 1) to 7.3 log_10_ TCID_50_/mL (passage 8) (data not shown). In order to evaluate the virulence of this virus, IC mice (n = 8) were infected with 2.1 × 10^4^ PFU/50 µl of lung homogenate from one mouse of passage 8 resulting in 75% mortality between days 6 and 7 p.i. (data not shown). These results indicate that the A/Switzerland/9715293/2013 isolate had acquired mutations that significantly affected virulence in mice. This adapted virus was called MA-H3N2.

### Kinetics of replication of parental and mouse-adapted H3N2 viruses *in vitro*

**Figure 2:**
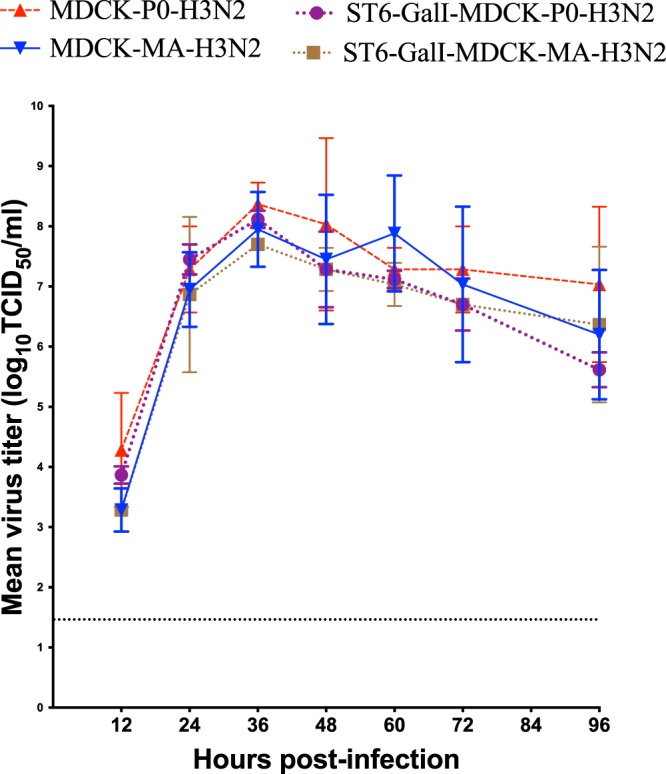
Kinetics of replication of parental (P0-H3N2) and mouse-adapted (MA-H3N2) A/H3N2 viruses in MDCK and ST6-GalI-MDCK cells. Confluent cells were infected with each of the viruses at a multiplicity of infection (MOI) of 0.0001 PFU/cell. Supernatants were harvested at 12, 24, 36, 48, 72, and 96 h postinfection and titrated by end point titration in 96-well plates. Titers are expressed in log_10_ TCID50/ml. The dashed horizontal line indicates the lower limit of detection.

To evaluate the replicative ability of MA-H3N2, virus yields assays were performed comparing the adapted virus with the parental virus (P0-H3N2) after multiple replication cycles, in both MDCK and ST6-GalI-MDCK cells (Fig. 2). *In vitro* growth kinetics revealed that both the P0-H3N2 and MA-H3N2 viruses achieved similar titers at all time points in both cell lines. Both parental virus and mouse-adapted strains generated peak titers at 36 h p.i. (Fig. 2). The mean peak viral titers of P0-H3N2 and MA-H3N2 viruses were 8.4 and 7.95 log_10_ TCID_50_/mL in MDCK cells and 8.1 and 7.7 log_10_ TCID_50_/mL in ST6-GalI-MDCK cells, respectively. These data show that the enhanced virulence of the MA-H3N2 virus in mice does not correlate with *in vitro* replication in standard cell lines for influenza viruses.

### Virulence and kinetics of replication of P0-H3N2 and MA-H3N2 viruses in IS and IC mice

The virulence of the generated MA-H3N2 variant was compared to that of the parental virus (P0-H3N2), in two C57BL/6 mouse models i.e., IC and pharmacologically-induced IS mice.

**Figure 3:**
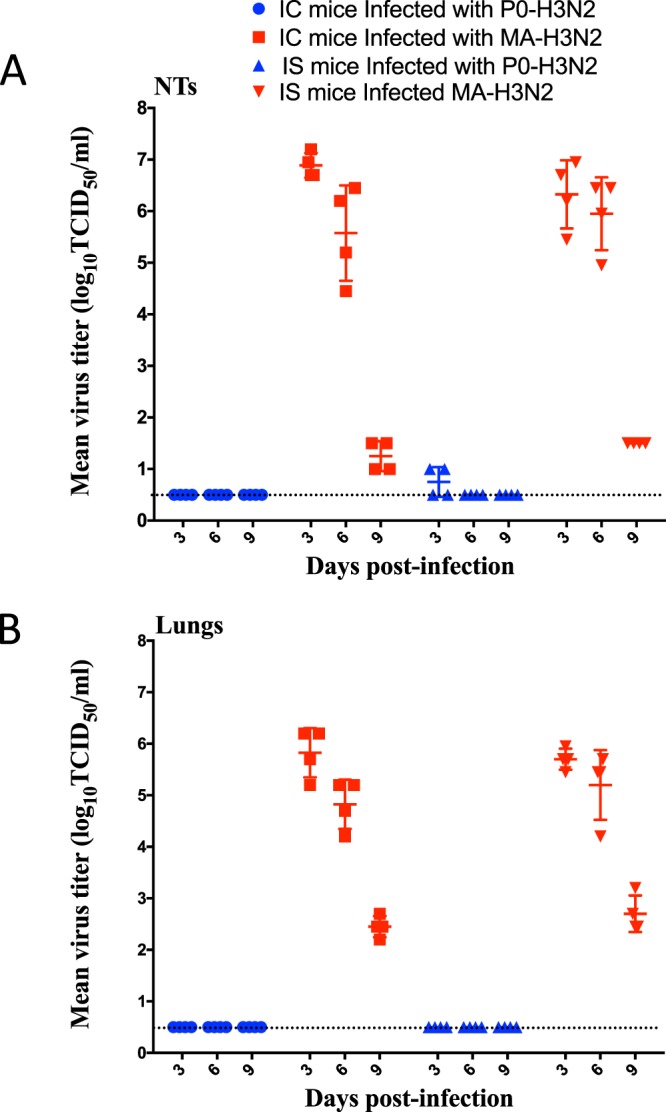
Kinetics of replication of P0-H3N2 and MA-H3N2 viruses in IC and IS mice. Group of mice (n = 5) were intranasally infected with 1 × 10^4^ PFU/mouse. Virus titers in the nasal turbinates (**A**) and lungs (**B**) of 5 mice per group sacrificed on 3 and 6 dpi are expressed as log_10_ TCID_50_/mL of tissue. Horizontal bars represent mean titers and symbols represent titers from individual mice. The dashed horizontal line indicates the lower limit of detection.

We observed that the MA-H3N2 virus was significantly more virulent than the parental virus in both IC and IS mice with a 50% mouse lethal dose (MLD_50_) of 3.6 and 2.0 × 10^3^ PFU, respectively, compared to no mortality for the parental virus (see Supplementary Figure). The kinetics of replication for a given dose of 1 × 10^4^ PFU/mouse of P0-H3N2 and MA-H3N2 viruses in IC and IS mice are displayed in Fig. 3. MA-H3N2 virus replicated to high titers in both the upper and lower respiratory tracts. Virus titers in the nasal turbinates (NTs) of IC and IS mice inoculated with MA-H3N2 virus peaked at 10^6.8^ and 10^6.3^ TCID_50_/mL, respectively, at 3 days post infection (dpi) while P0-H3N2 did not replicate at all in IC mice and exhibited very low titers in the NTs of IS mice (10^0.75^ TCID_50_/mL) (Fig. 3A). A similar pattern was observed in the lungs of IC and IS mice with virus titers of 10^5.8^ and 10^5.7^ TCID_50_/mL, respectively, at 3 dpi. Detectable virus lung titers were still observed at day 9 p.i. in both mouse models infected with MA-H3N2 virus (10^2.45^ and 10^2.7^ TCID_50_/mL for IC and IS mice, respectively). P0-H3N2 did not replicate at all in the lungs of mice with an inoculum of 1 × 10^4^ PFU/mouse (Fig. 3B). None of the viruses, P0-H3N2 or MA-H3N2, were isolated in the brain or spleen of IC or IS mice (data not shown).

### Whole genome sequence analysis

We sequenced the genomes of the parental, an intermediate virus isolated after 7 passages in IS mice (P7-IS-H3N2) and the mouse-adapted H3N2 viruses to identify the amino acid substitutions responsible for the increased virulence in mice (Table 1). Compared to the genetic sequences of the parental virus, amino acid substitutions were identified in four protein-coding regions in both P7-IS-H3N2 and MA-H3N2 viruses. Specifically, both passaged viruses harbored mutations in PA (K615E), HA (N144E, N246K and A304T), NP (G384R) and NA (G320E). An additional amino acid mutation in the HA protein (N122D) was detected in the MA-H3N2 virus.

**Table 1:** Amino acid substitutions between of P0-H3N2, P7-IS-H3N2* and MA-H3N2 viruses.

Virus protein	P0-H3N2	Mutation sites (residue number)	
		P7-IS-H3N2	MA-H3N2
PB2	—	—	—
PB1	—	—	—
PA	K615	615E	615E
HA	N122	—	122D
	N144	144E	144E
	N246	246 K	246K
	A304	304 T	304T
NP	G384	384 R	384 R
NA	G320	320E	320E
M	—	—	—
NS	—	—	—

*P7-IS-H3N2: virus isolated after 7 passages in immunosuppressed mice.

### Cytokine and chemokine analysis

**Figure 4:**
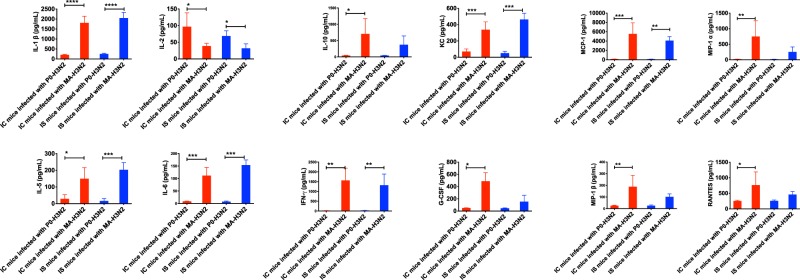
Pulmonary cytokines/chemokine levels on day 6 post-infection in IC and IS C57BL/6 mice intranasally infected with 1 × 10^4^ PFU/mouse of P0-H3N2 and MA-H3N2 viruses. Bars represent mean values ± SDs (n = 4/group). One-way analysis of variance (ANOVA) with Tukey’s multiple comparison post-test (*p < 0.005, **p < 0.01, ***p < 0.001, ****p < 0.0001) was used for the comparisons.

We evaluated and compared 23 cytokines and chemokines in lung homogenates of IC and IS mice infected with both P0-H3N2 and MA-H3N2 viruses on day 6 p.i. (Fig. 4). In IC mice, MA-H3N2 virus induced statistically significant increases in IL-1β, IL-5, IL-6, IL-10, KC, IFN-γ, G-CSF, MCP-1, MIP-1α, MIP-1β, and RANTES levels compared to the P0-H3N2 virus. Only IL-2 levels were significantly lower in MA-H3N2-infected IC mice. IS mice infected with the MA-H3N2 virus had significant increases in IL-1β, IL-5, IL-6, KC, IFN-γ, and MCP-1 levels and significant decrease of IL-2 level compared to the P0-H3N2 virus (Fig. 4). No changes in the levels of the other cytokines (IL-1α, IL-2, IL-3, IL-4, IL-6, IL-9, IL-12(p40), IL-12(p70), IL-13, IL-17A, eotaxin, GM-CSF and TNF-α) were observed between the two groups of mice (data not shown).

### Histopathology

**Figure 5:**
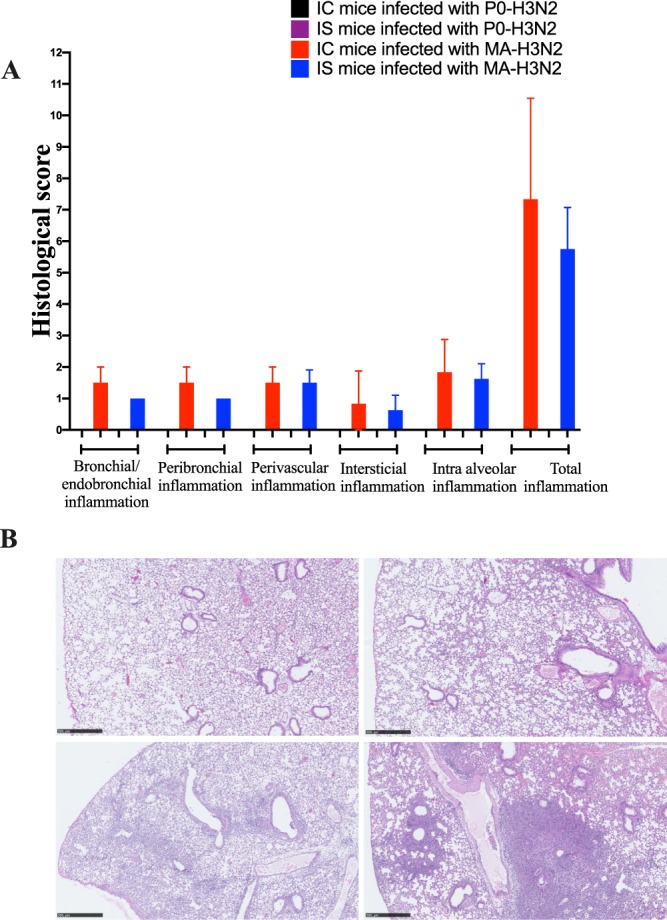
Lung histopathology on day 6 post-infection in IC and IS C57BL/6 mice intranasally infected with 1 × 10^4^ PFU/mouse of P0-H3N2 and MA-H3N2 viruses. (**A**) Histological scores of IC and IS mice. (**B**) Pulmonary histology of IS mice infected with P0-H3N2 (upper left; inflammation score 0), IS mice infected with MA-H3N2 (lower left; inflammation score 7), IC mice infected with P0-H3N2 (upper right; inflammation score 0) and IC mice infected with MA-H3N2 (lower right; inflammation score 11). All photomicrographs are from 4 µm thick sections of formalin-fixed paraffin-embedded lung tissue stained with hematoxylin-eosin scanned with a Nanozoomer 2.0 (Hamamatsu, Japan). Micrometric scale of 500 µm.

To confirm the pathogenicity of the MA-H3N2 virus, we investigated the histopathological changes in lungs of IC- and IS- infected mice at day 6 p.i. There was no lung damage in IC or IS mice infected with P0-H3N2 (total inflammation score of 0) (Fig. 5A). In contrast, total inflammation scores in the lungs of IC and IS mice infected with the MA-H3N2 virus were significantly increased (*p* < 0.0001) compared to IC and IS mice infected with P0-H3N2 virus. In general, IC mice infected with the MA-H3N2 virus had higher inflammation scores in all evaluated parameters, although the differences for single parameters were not significant. Similarly, the total inflammation score for IC mice infected with MA-H3N2 virus was higher (7.3 ± 2.0) than that of the IS mice infected with the same virus (5.7 ± 1.1), although the difference was not significant. Figure 5B shows representative lung tissues stained with hematoxylin-eosin for both viruses in IC and IS mice.

### Generation of recombinant H3N2 influenza viruses

Seven amino acid substitutions were identified in the PA (K615E), NP (G384R), NA (G320E) and HA (N122D, N144E, N246K, and A304T) proteins of MA-H3N2 compared to the parental virus (Table 1). To identify the viral factors that contributed to mouse-adaptation, we used site-directed mutagenesis and reverse genetics in order to rescue several recombinant (rec) viruses containing one or more of the MA-H3N2 associated segments with the remaining segments from the parental virus (P0-H3N2) (Table 2). The rec viruses were named according to the origin of their exchanged genes. For example, the rec virus containing the PA derived from MA-H3N2 with the rest of its genes from P0-H3N2 was named rec MA-H3N2-PA.

**Table 2:** Schematic representation of recombinant viruses generated with different plasmid combinations.

Viruses	Genotype							
	PB2	PB1	PA	NP	HA	NA	M	NS
rec P0-H3N2								
rec MA-H3N2			*	*	*	*		
rec MA-H3N2-PA			*					
rec MA-H3N2-NP				*				
rec MA-H3N2-HA					*			
rec MA-H3N2-NA						*		
rec MA-H3N2-(PA + NP)			*	*				
rec MA-H3N2-(PA + HA)			*		*			
rec MA-H3N2-(PA + NA)			*			*		
rec MA-H3N2-(NP + HA)				*	*			
rec MA-H3N2-(NP + NA)				*		*		
rec MA-H3N2-(HA + NA)					*	*		
rec MA-H3N2-(PA + NP + HA)			*	*	*			
rec MA-H3N2-(PA + NP + NA)			*	*		*		
rec MA-H3N2-(PA + HA + NA)			*		*	*		
rec MA-H3N2-(NP + HA + NA)				*	*	*		
rec MA-H3N2-(PA_615E_ + HA_144E_)			* 615E		* 144E			
rec MA-H3N2-(PA_615E_ + HA_246K_)			* 615E		* 246K			
rec MA-H3N2-(PA_615E_ + HA_304T_)			* 615E		* 304T			
rec MA-H3N2-(PA_615E_ + HA_144E+246K_)			* 615E		*144E + 246 K			
rec MA-H3N2-(PA_615E_ + HA_144E+304T_)			* 615E		*144E + 304 T			
rec MA-H3N2-(PA_615E_ + HA_246K+304T_)			* 615E		*246 K + 304 T			

### Replication kinetics of recombinant viruses *in vitro*

**Figure 6:**
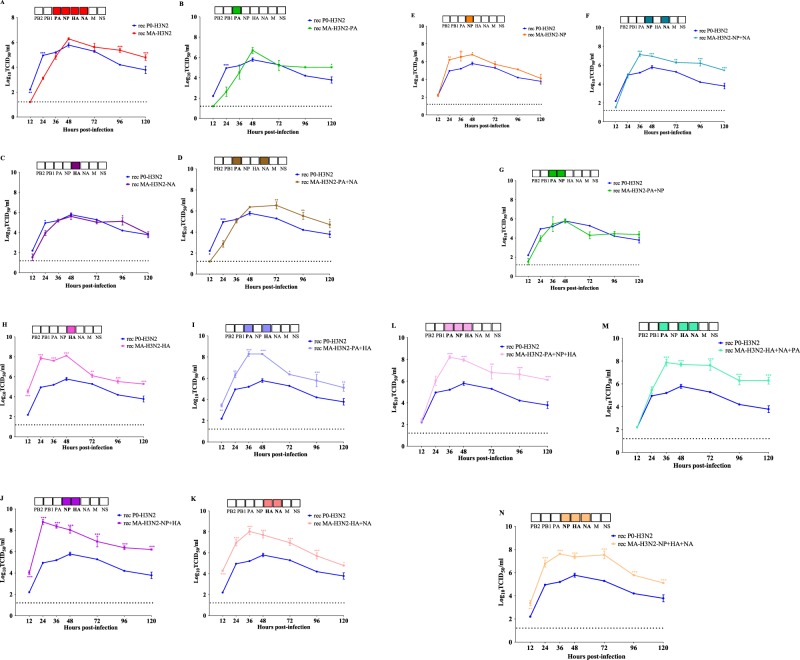
Replicative capacity of P0-H3N2 and recombinant influenza viruses in STG6GalI-MDCK cells. Cells were infected at a MOI of 0.0001 PFU/cell in triplicate for each group and rec viruses were harvested at 12, 24, 36, 48, 72, 96, and 120 h postinfection and titrated by end point titration in 96-well plates. Titers are expressed in log_10_ TCID50/ml. The mean values for three experiments with standard deviations are presented. *P < 0.05, **P < 0.01, ***P < 0.001. The dashed horizontal line indicates the lower limit of detection.

To explore the effect of mouse-adapted substitutions on growth properties in mammalian cells, we performed replication kinetics experiments using MDCKα2.6 cells. As shown in Fig. 6A, the rec-P0-H3N2 virus grew at significantly higher titers at 12 and 24 hours post infection (hpi) compared to rec MA-H3N2. However, the rec P0-H3N2 and rec MA-H3N2 viruses had similar viral titers at 36, 48, and 72 hpi. Thereafter, at 96 and 120 hpi, the rec MA-H3N2 showed higher titers. Replacement of the PA or NA segments in the P0-H3N2 with those of the MA-H3N2 virus did not alter the replication kinetics (Fig. 6B,C). Of note, rec MA-H3N2-PA + NA virus had significant higher titers at 72, 96, and 120 hpi than the rec P0-H3N2 virus (Fig. 6D). Interestingly, the rec MA-H3N2-NP virus had significantly higher titers at 24, 36 and 48 hpi than the rec P0-H3N2 virus (Fig. 6E). When MA-H3N2-NP and NA segments were combined, significant higher titers were also observed at 36–120 hpi (Fig. 6F). By contrast, no significant increase in viral titers was seen with the combination MA-H3N2-PA and NP segments (Fig. 6G). Noteworthy, rec viruses containing the MA-H3N2-HA alone (Fig. 6H) or in combination with other mutated genes (Fig. 6I–N) exhibited higher replication efficiency compared to the rec P0-H3N2 virus at several times points. Collectively, these finding highlights the importance of the MA-H3N2-HA gene for increased replication *in vitro*.

### Minigenome assay for polymerase activity

**Figure 7:**
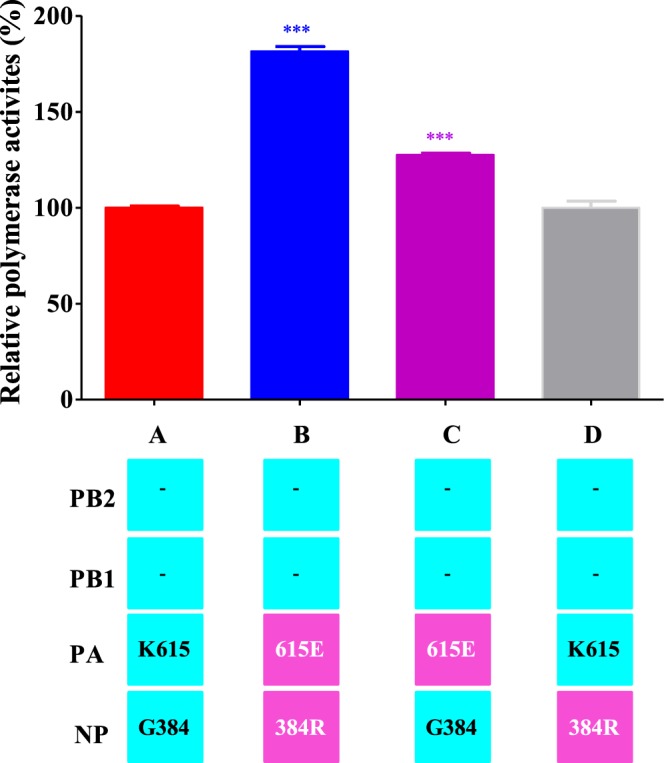
Polymerase activity of RNP complex combinations between the P0-H3N2 and MA-H3N2 viruses by minigenome assay. Each luciferase activity value is the average of three independent experiments performed in triplicate and is normalized to the average of luciferase values for the three separate P0-H3N2 replicates which was set to 100% as measured on 293 T cells. The PB2 and PB1 genes had no mutations relative to P0-H3N2 (indicated by dashes). The gene segments derived from P0-H3N2 and MA-H3N2 are shown in blue and pink, respectively. ***P < 0.0001 compared to P0-H3N2.

To examine whether the observed mutations in the PA and NP proteins of MA-H3N2 virus affected the transcription activity of the ribonucleoprotein (RNP) complex, we performed a luciferase minigenome assay in 293 T cells. The polymerase activity of rec virus containing the PA-615E and NP-384R mutations was significant higher (181.5%; P < 0.0001) than that of rec P0-H3N2 parental virus (100%) (Fig. 7), reflecting higher transcription and replication activity. To assign this increased activity to a specific polymerase gene, an analysis of two combinations of the polymerase subunits was performed. A significant higher level (127.6%; P < 0.0001) of luciferase activity was observed with PA-615E subunit compared to that of the rec P0-H3N2 virus, whereas the transcription activity of NP-384R subunit had no significant effect, reflecting the predominant implication of MA-H3N2-PA gene in the increased polymerase activity in mammalian cells (Fig. 7).

### Identification of substitutions involved in increased virulence in mice

**Figure 8:**
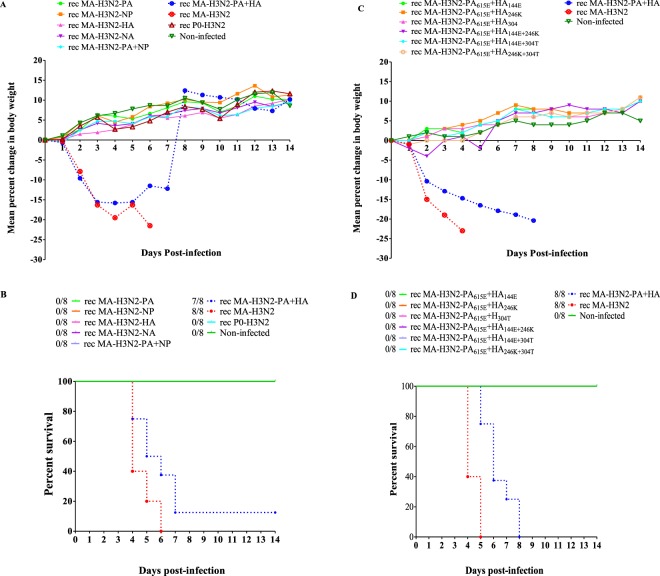
Virulence of rescued recombinants viruses in C57BL/6 mice. (A,B) Body weight loss and survival curve of mice inoculated with 1 × 10^5^ PFU/_50µl_ of each recombinant virus. (C,D) Body weight loss and survival curve of mice infected with 1 × 10^5^ PFU of recombinant viruses containing a single substitution (K615E) in MA-H3N2-PA segment and various substitutions in MA-H3N2-HA segment (N144E, N246K, and A304T). Mice were observed daily for 14 days for clinical signs of illness, including weight loss, ruffled fur, and hunching and were sacrificed if they lost ≥20% of their original body weight.

To determine the genetic basis for the high virulence of the rec MA-H3N2, groups of mice were infected intranasally with 1 × 10^5^ PFU of each rec virus and monitored daily for body weight loss and lethality. All mice infected with the rec MA-H3N2 died by 6 dpi. Mice inoculated with rec P0-H3N2 viruses containing only one mutated segment (rec MA-H3N2-PA, rec MA-H3N2-HA, and rec MA-H3N2-NP) or a combination of two mutated segments (rec MA-H3N2-PA + NP) did not die over 14 days p.i. The rec MA-H3N2 and rec MA-H3N2-PA + HA group suffered the most drastic weight loss, 21.5% and 16.1% by 6 and 5 dpi, respectively (Fig. 8A). Mice infected with the rec MA-H3N2-PA + HA virus exhibited 87.5% of mortality (Fig. 8B) suggesting a synergic effect between MA-PA and MA-HA mutations on virulence in mice. To analyze the impact of each single and/or double adaptive mutations from MA-HA and MA-PA on virulence, we rescued several rec viruses (referred as; rec MA-H3N2-PA_615E_ + HA_144E_, rec MA-H3N2-PA_615E_ + HA_246K_, rec MA-H3N2-PA_615E_ + HA_304T_, rec MA-H3N2-PA_615E_ + HA_144E+246K_, rec MA-H3N2-PA_615E_ + HA_144E+304T_, and rec MA-H3N2-PA_615E_ + HA_246K+304T_) (Table 2). Of note, rec MA-H3N2-HA_122D_ was not evaluated because in a previous study in mice infected with 1 × 10^5^ PFU/50 µl of P7-IS-H3N2 virus, the latter demonstrated a virulence similar to MA-H3N2 (data not shown). Since the only difference in the genome of the two viruses was the HA-N122D substitution, it could be concluded that this mutation was not responsible for the adaptation. Thus, eight groups of C57BL/6 mice were infected with 1 × 10^5^ PFU of each mutant, and monitored for 14 days. We did not observe significant weight loss after combining one or two MA-HA mutations with the single MA-H3N2-PA mutation (Fig. 8C). However, all rec MA-H3N2-PA + HA infected mice died by 8 dpi (100% mortality) (Fig. 8D). These results demonstrate that both MA-H3N2-PA containing one mutation (K615E) and MA-H3N2-HA containing all 3 mutations (N144E, N246K, and A304T) are required for the increased pathogenicity of MA-H3N2 virus in mice.

## Discussion

Seasonal H3N2 viruses do not replicate well in mice. Historically, old mouse-adapted A/H3N2 variants, such as A/Hong Kong/1/68, A/Aichi/2/68, and A/Victoria/3/75, have been frequently used to evaluate the activities of new antivirals drugs or vaccine^36–38^. The objective of this study was to generate a contemporary mouse-adapted H3N2 strain and to identify the molecular basis for viral adaptation in this species. We selected a recent A/H3N2 influenza strain (A/Switzerland/9715293/2013) and generated a pathogenic mouse-adapted virus (MA-H3N2) after fifteen serial lung-to-lung passages in mice. As expected, mice infected with the wild-type virus (P0-H3N2) did not exhibit clinical signs of illness or death. In contrast, more serious body disease resulting in weight loss, significant mortality, high virus titers, and more severe pathological changes in lung tissues were observed in the group of mice infected with the MA-H3N2 virus.

Genomic analysis indicated that the MA-H3N2 variant acquired seven amino acid substitutions among four gene segments, PA(K615E), NP(G384R), HA(N144E, N144D, N246K, and A304T), and NA(G320E). The RNP complexes containing the PA(K615E) presented significant higher transcription activity in 293 T cells compared to the parental virus while this single mutant did not influence replication kinetics in ST6-GalI-MDCK cells and pathogenicity in mice, indicating that adaptation requires additional mutations. In recent years, studies have shown that multiple amino acid substitutions in the PA gene such as PA-T20A, K22R, T97I, M155T, D216N, P277S, L315F, P355S, and K615N/R, have been associated with adaptation in mice^17,18,23^. Gabriel *et al*.^17^ reported that amino acid substitutions at residue 615 were associated with the adaptation of H5N2 and H7N7 influenza viruses by enhancing the polymerase activity and the virulence in mice together with other mutations^17,23,39^. These results suggest that the PA(K615E) substitution in MA-H3N2 may play a similar role to that of previously identified mutations at position 615 in the PA protein.

The HA protein is responsible for the initial attachment of the influenza virus to the host cell membrane by binding to sialic acid (SA) receptors, linked to galactose through either SAα2–3 or SAα2–6 bonds^40^. *In vitro*, the HA (N122D, N144D, N246K, and A304T) mutants displayed the highest replication kinetics in ST6-GalI-MDCK cells, but these mutations alone did not correlate with increased pathogenicity in mice. The HA (N144E and N246K) substitutions found in the MA-H3N2 virus potentially abolished N-linked glycosylation sites. In an experimental approach, removal of N-glycosylation sites from influenza virus led to increased pathogenicity to mice^41^. These observations suggest that the loss of N-linked glycosylation may play a critical role in increasing the virulence to mice. Previous reports have shown that A/H3N2 viruses with a loss of N-linked glycosylation sites at residues 144 and 246 are associated with significant increased pathogenicity *in vivo*, which translated in higher viral replication and greater pulmonary inflammation^42–44^. Bragstad *et al*.^45^ suggested that the loss of glycosylation at residue 144 in the HA protein might have contributed to the enhanced infectivity of the reassorted A/H3N2 viruses during the 2003–2004 season in Denmark. The HA (A304T) mutation was not reported in previous studies and its functional role in the enhanced virulence of rec MA-H3N2 is unknown, but our study showed that this mutation is necessary to increase the virulence in mice. It has been suggested that the gain or/and the loss of N-linked glycosylation sites in the HA protein has selective advantages to the influenza virus by preventing the binding of antibodies to antigenic sites and also participate in the antigenic drift. For instance, Skehel *et al*. have previously reported that a gain of N-glycosylation site at position 63 in HA1 allowed an antigenic variant of an H3N2 to escape neutralization by a monoclonal antibody (MAbs)^46^. Gu *et al*., reported that a gain of N-glycosylation at position 131 and an amino acid insertion at position 134 in the HA protein eliminated the reactivity of H5N1 viruses with the MAbs, and therefore, have a role in the antigenic variation of influenza viruses^47^. We have performed hemagglutination inhibition assay (HAI) (data not shown) with the P0-H3N2 and MA-H3N2 viruses against serum from mice inoculated with the P0-H3N2 virus and observed no significant difference in the HAI titers (80 and 160, respectively.) Therefore, we conclude that our adapted virus did not alter the antigenicity of the H3N2 strain and could be used for vaccine challenge studies.

Overall, the results obtained in the present study are in line with those obtained in previous studies^42–45^ where the loss of N-linked glycosylation sites on the HA protein of MA-H3N2 influenza virus is a critical factor modulating the virulence and pathogenicity in mice. Collectively, our data reveal that the MA-H3N2-PA and MA-H3N2-HA genes play critical roles in pathogenicity of our adapted virus. The rec viruses containing either the MA-H3N2-HA or MA-H3N2-PA segment alone were not as virulent in mice while the rec virus containing both segments (PA and HA) had similar virulence to rec MA-H3N2. Therefore, the PA (K615E) and HA (N144E, N246K, and A304T) are necessary for adaptation and acquisition of the virulence in mice. Furthermore, the evaluation of the effect of individual or double substitutions in the HA protein in combination with PA mutation does not appear to affect the virulence and pathogenicity in mice compared to rec virus possessing both segments with all mutations (rec MA-H3N2-PA + HA). Thus, our findings suggest that the substitutions in the PA (K615E) and HA (N144E, N246K, and A304T) proteins are all required for a virulent phenotype of the MA-H3N2 in mice.

Further, we found that infection with the MA-H3N2 virus induced high levels of pro-inflammatory cytokines and chemokines in NTs and lungs of mice. Previous data suggested that cytokine/chemokine production is closely related to the host damage after influenza virus infection^48^. Indeed, we detected significantly higher levels of IL-5, IL6, IL-10, INF-γ, IL-1β, KC, G-CSF, MCP-1, MIP-α, MIP-1β, and RANTES in lungs of IC mice and IL-1β, IL-5, IL-6, KC, IFN-γ, and MCP-1 levels in lungs of IS mice infected with the MA-H3N2 virus compared to those infected with the P0-H3N2 virus. These results suggest that the MA-H3N2 mutations may enhance the ability of the virus to replicate in mouse lungs, which is positively correlated with weight loss and high lungs viral titers at day 6 p.i. High levels of IL-6 have been observed to correlate with infection severity while IL-5 levels were associated with a delay in influenza virus clearance in infected mice^49–51^. IFN-γ was shown to mediate the increased production of nitric oxide which can subsequently result in the recruitment of more neutrophils and macrophages^52^. IL-10 is a negative regulator of inflammation, and high levels of this cytokine may act as a feedback regulator of virally-induced severe inflammation^53^. High concentrations of MCP-1 were observed to be responsible for recruiting inflammatory cells in the lungs of mice infected with highly pathogenic viruses^54–56^. High G-CSF levels were observed in lungs and sera from mice infected with A/H3N2 followed by superinfection with Streptococcus pneumoniae serotype 3, leading to fatal disease^57^. High levels of MCP-1, MIP-1α, MIP-1β, RANTES, and KC have been strongly upregulated during infection with the A/H5N1 virus, leading to the recruitment of neutrophils and monocytes in the lung and resulting in acute inflammation correlated with severe pulmonary disease^48^. Furthermore, our results show that the MA-H3N2 virus clearly elicited robust inflammatory response, which may explain the extensive pathological lesions, including hyperaemia, edema, and exudative pathological changes in the lung tissues compared to the wild-type virus.

For influenza virus, the efficacy of therapeutic agents has been extensively evaluated in mice by challenging with lethal adapted variants^58–60^. Our mouse-lethal A/H3N2 virus is an important preclinical tool to test the efficacy of antiviral drugs options for A/H3N2 variant, with the aim to improve the treatment outcomes in both immunocompetent and immunosuppressed patients. Furthermore, a recombinant virus generated by reverse genetics provides a powerful technology to develop influenza virus vaccines and to study the mechanisms of antiviral resistance, fitness, and pathogenesis.

In summary, we generated a lethal mouse model of seasonal A/H3N2 virus following serial lung-to-lung passages of a wild-type virus in mice and found that both HA (N144E, N246K, and A304T) and PA (K615E) genes co-contribute to enhance viral pathogenicity and virulence in mice. This present study suggests that adaptation and virulence of the MA-H3N2 in mice are clearly polygenic as identified in other studies^61–63^. Our mouse-lethal A/H3N2 seasonal virus can be used for evaluation of new antiviral agents and vaccine candidates.

## Methods

### Ethics statements

This study was conducted in agreement with the guidelines of the Canadian Council on Animal Care. All experimental protocols were approved by the Institutional Animal Care Committee at Université Laval.

### Cells and virus

Madin-Darby canine kidney (MDCK) were obtained from the American Type Culture Collection (ATCC: CCL-34) (Manassas, VA, USA) and were grown in minimum essential medium (MEM) Supplemented with 10% fetal bovine serum (FBS, Invitrogen), HEPES and antibiotics. MDCK cells overexpressing the α2.6 sialic acid receptor (ST6-GalI-MDCK cells) were kindly provided by Y. Kawaoka from the University of Wisconsin, Madison, WI^64^, and grown in MEM supplemented with 10% FBS, HEPES and 7.5 μg/ml of puromycin. The human embryonic kidney 293 T cell line (ATCC, CRL-3216) was maintained in Dulbecco’s modified Eagle’s medium (DMEM, Invitrogen), supplemented with 10% FBS and HEPES. The influenza A/Switzerland/9715293/2013 (A/H3N2/sw/2013) virus was obtained from NIBSC (code number 14/224).

### Mouse adaptation

Six-to eight-week-old female C57BL/6 mice were purchased from Charles River Canada (St-Constant, Quebec, Canada). Animals were housed 3 per cage, kept under conditions which prevented cage-to-cage infections and fed with sterilized food and water. A mouse-adapted variant was derived from a series of sequential lung-to-lung passages in pharmacologically-induced IS mice followed by sequential lung-to-lung passages in IC mice. Briefly, mice (n = 3/passage) were treated intraperitoneally (i.p.) with 100 mg/kg of cyclophosphamide (CP; Sigma, St-Louis. MO) one day before infection and on day 3 post-infection (p.i.)^32,65^. Fifty μL of MEM containing 2.5 × 10^5^ plaque-forming units (PFU)/mouse of A/H3N2/sw/2013 virus (passage 1) were inoculated intranasally (i.n.). At four days p.i., three inoculated mice were euthanized by cervical dislocation under isoflurane anesthesia and lungs were harvested and homogenized in 1 mL of PBS containing 2X antibiotic-antimycotic solution (penicillin streptomycin and amphotericin B) (Invitrogen-Gibco) using the Omni Tip^TM^ Homogenizer. Tissue homogenates were clarified by centrifugation (2000 x g for 5 min), then supernatants were collected and 50 μL of the centrifuged homogenate were used to infect the next three naïve mice (passage 2) the same day. After 7 passages, the virus in the lung homogenates was cloned once by plaque purification in ST6-GalI-MDCK cells and then amplified and titrated in the same cell line. An inoculum of 2.5 × 10^4^ PFU/mouse was used to perform 8 additional lung-to-lung passages in IC mice. Lung homogenates were titrated in ST6-GalI-MDCK cells by end-point titration in 96-well plates. Briefly, 10-fold serial dilutions were made and 20 µl/well of virus were added to confluent cells plates were incubated at 37 °C, 5% CO_2_ for 4 days. Titers were recorded by the presence of cytopathic effects and expressed as log_10_ of the mean tissue culture infectious dose per mL (TCID_50_/mL). The virus producing the highest lung homogenate titer, so-called MA-H3N2, was plaque purified and amplified in ST6-GalI-MDCK cells.

### Kinetics of replication of parental, adapted and recombinant H3N2 viruses *in vitro*

Confluent MDCK and ST6-GalI-MDCK cells were infected with the parental H3N2 (P0-H3N2) and the adapted H3N2 (MA-H3N2) viruses at a multiplicity of infection (M.O.I.) of 0.0001 PFUs/cell. Supernatants were collected at 12, 24, 36, 48, 72 and 96 hpi. Virus titers were determined by end point titration as described above and expressed as log_10_ TCID_50_/mL. The kinetics of replication of recombinant H3N2 viruses were performed as described above but supernatants were collected at 12, 24, 36, 48, 72, 96 and 120 hpi.

### Virulence and kinetics of replication of P0-H3N2 and MA-H3N2 viruses in IS and IC mice

Groups of five six-to eight-week-old female IS or IC C57BL/6 mice were inoculated with 10^1^ to 10^5^ PFU/50 µl of P0-H3N2 or 10^2^ to 10^5^ PFU/50 µl of MA-H3N2 viruses, respectively. Mice were observed daily for 14 days for clinical signs of illness, including weight loss, ruffled fur, and hunching. In accordance with our animal study protocol, mice were sacrificed if they lost ≥20% of their original body weight. The 50% mouse lethal dose (MLD_50_) was calculated for lethal viruses using the Reed and Muench method^66^. To determine lung titers, groups of 12 mice were infected with 1 × 10^4^ PFU/mouse of P0-H3N2 and MA-H3N2 viruses. Four mice per group were sacrificed on days 3, 6 and 9 p.i. and lungs, NTs, spleens and brains were harvested and stored at −80 °C. Organs were homogenized in 1 mL of PBS containing 2X antibiotic-antimycotic solution as described above. Tissue homogenates were clarified by centrifugation (2000 x g for 5 min) and supernatants were titrated in ST6-GalI-MDCK cells. Titers were expressed as log_10_ TCID_50_/mL.

### Whole genome sequence analysis

Viral RNA was extracted from 140 µl of lung homogenates using the QIAamp Viral RNA kit (Qiagen) and eluted in 100 µL of elution buffer. cDNA was synthesized using random hexamer primers (Amersham Pharmacia Biotech) and the SuperScript II reverse transcriptase enzyme (Life Technologies Corporation). Viral cDNAs were amplified by PCR using the Phusion high-fidelity DNA polymerase (New England BioLabs, Whitby, ON, Canada) and universal primers^67^ in standard conditions. The nucleotide sequences of the PCR products were determined using the ABI 3730 DNA analyzer, and chromatogram peaks were analyzed using BioEdit, version 7.0.5.

### Cytokine and chemokine analysis

The concentration of 23 cytokines and chemokines (IL-1α, IL-1β, IL-2, IL-3, IL-4, IL-5, IL-6, IL-9, IL-10, IL-12(p40), IL-12(p70), IL-13, IL-17A, eotaxin, G-CSF, GM-CSF, IFN-γ, KC, MCP-1, MIP-1α, MIP-1β, RANTES and TNF-α) were measured on day 6 p.i. in lung homogenates (n = 4/group) from both IC and IS mice infected with P0-H3N2 or MA-H3N2 viruses using the Bio-Plex Pro^TM^ Mouse Cytokine 23-plex panel (Bio-Rad Laboratories) according to the manufacturer’s instructions. Cytokine and chemokine concentrations were expressed as pg/ml of lung.

### Histopathology

To compare the lung histopathology induced by P0-H3N2 and MA-H3N2 viruses in IC and IS mice, four mice per group were anesthetized and intranasally infected with 1 × 10^4^ PFU/mouse of P0-H3N2 and MA-H3N2 viruses. At 6 days p.i., mice were euthanized and their lungs were fixed in 4% paraformaldehyde, embedded in paraffin and cut into four μm-thick histologic sections. Sections were then stained with hematoxylin and eosin. Slides were digitalized at 40X magnification using a Nanozoomer slide scanner (Hamamatsu, Japan) and scored using NDP viewer 2.0 software (Hamamatsu, Japan). The histopathological scores were determined by a pathologist and a medical biologist who were blinded to the experimental data. A semi-quantitative scale was used to score bronchial/endobronchial, peribronchial, perivascular, interstitial, pleural and intra alveolar inflammation, capillary vascular congestion and pulmonary edema^68^. The results were expressed as total pulmonary inflammatory scores.

### Generation of recombinant H3N2 influenza viruses

Reverse transcription-PCR using universal influenza primers^67^ was performed to amplify the eight genomic segments of P0-H3N2 and MA-H3N2 viruses. All segments were cloned into bidirectional expression/translation pLLBA/G vectors as previously described^69^. Wild-type and mutant viruses (Table 1) were rescued in a 6-well plate of co-cultured 293 T and MDCK cell mixtures (5 × 10^5^ cells of each cell line) following transfection of the corresponding eight viral plasmids (each containing 1 µg of plasmid) using Lipofectamine 2000 (Invitrogen, Carlsbad, CA), according to the manufacturer’s instructions. Transfection medium was removed after 6 h and replaced with DMEM containing 1 µg/ml TPCK-treated trypsin (Sigma-Aldrich, St. Louis, MO, USA). The supernatant was harvested after 72 h post-transfection, and used to inoculate ST6GalI-MDCK cells to prepare stocks for subsequent analyses. The resulting rescued viruses were fully sequenced to ensure the absence of undesired mutations, titrated by plaque assays in ST6GalI-MDCK cells and then stored at −80 °C until used. Mutations found in the MA-H3N2 virus (Table 1) were introduced into the plasmid constructs of P0-H3N2, using appropriate primers and the QuikChange site-directed mutagenesis kit (Stratagene, La Jolla, CA), according to the manufacturer’s instructions. The resulting plasmids were sequenced to ensure the absence of undesired mutations.

### Minigenome assay for polymerase activity

To compare the effect of PA-_MUT_ and NP-_MUT_ segments of the MA-H3N2 virus on viral RNA polymerase activity, a reconstituted minigenome assay with a reporter plasmid containing the Gaussia luciferase gene flanked by noncoding regions of the non-structural (NS) gene of influenza A/Quebec/147144/09 virus (PNS-Luc) was designed^69^. Briefly, reporter plasmid PNS-Luc (1 µg) was transfected into 293 T cells using Lipofectamine 2000 reagent (Invitrogen, Carlsbad, CA), together with 1 µg of each of the pLLB plasmids encoding PB2, PB1, PA and NP (wild type or mutated H3N2 viruses) in 6-well plates. After 6 h, the transfection medium was replaced with fresh medium. Mock transfections were performed with PNS-Luc alone. Forty-eight hours following transfection, cells were harvested and luciferase activity was measured with a multilabel plate reader (Victor; PerkinElmer, Waltham, MA), with an acquisition period of 1 second. Luciferase activities of the different samples were normalized to the average of three independent experiments values for the three P0-H3N2 replicates, which was set to 100% as measured on 293 T cells.

### Statistical analyses

Lung viral titers, cytokine/chemokine levels and polymerase activities were compared by one-way analysis of variance (ANOVA) with Tukey’s multiple-comparison post-test. Replicative capacity of recombinant viruses and histopathologic scores were analyzed with two-way ANOVA with Tukey’s multiple-comparison post-test. All analyses were done using GraphPad, version 8.

## Supplementary information

Supl. Figure
