# Supplementary material for: Synergistic PA and HA mutations confer mouse adaptation of a contemporary A/H3N2 influenza virus
Source: Sci Rep. 2019 Nov 12;9:16616. doi: 10.1038/s41598-019-51877-4 (PMC6851088; doi:10.1038/s41598-019-51877-4)
Supplement: Supplementary file 1 — Supl. Figure [file 41598_2019_51877_MOESM1_ESM.pdf]

Synergistic PA and HA mutations confer mouse adaptation of a contemporary A/H3N2 influenza virus

Mariana Baz[#*1], Zeineb M'hamdi[*1], Julie Carbonneau[1], Sophie Lavigne[2], Christian Couture[2], Yacine Abed[1] and Guy Boivin[#1]

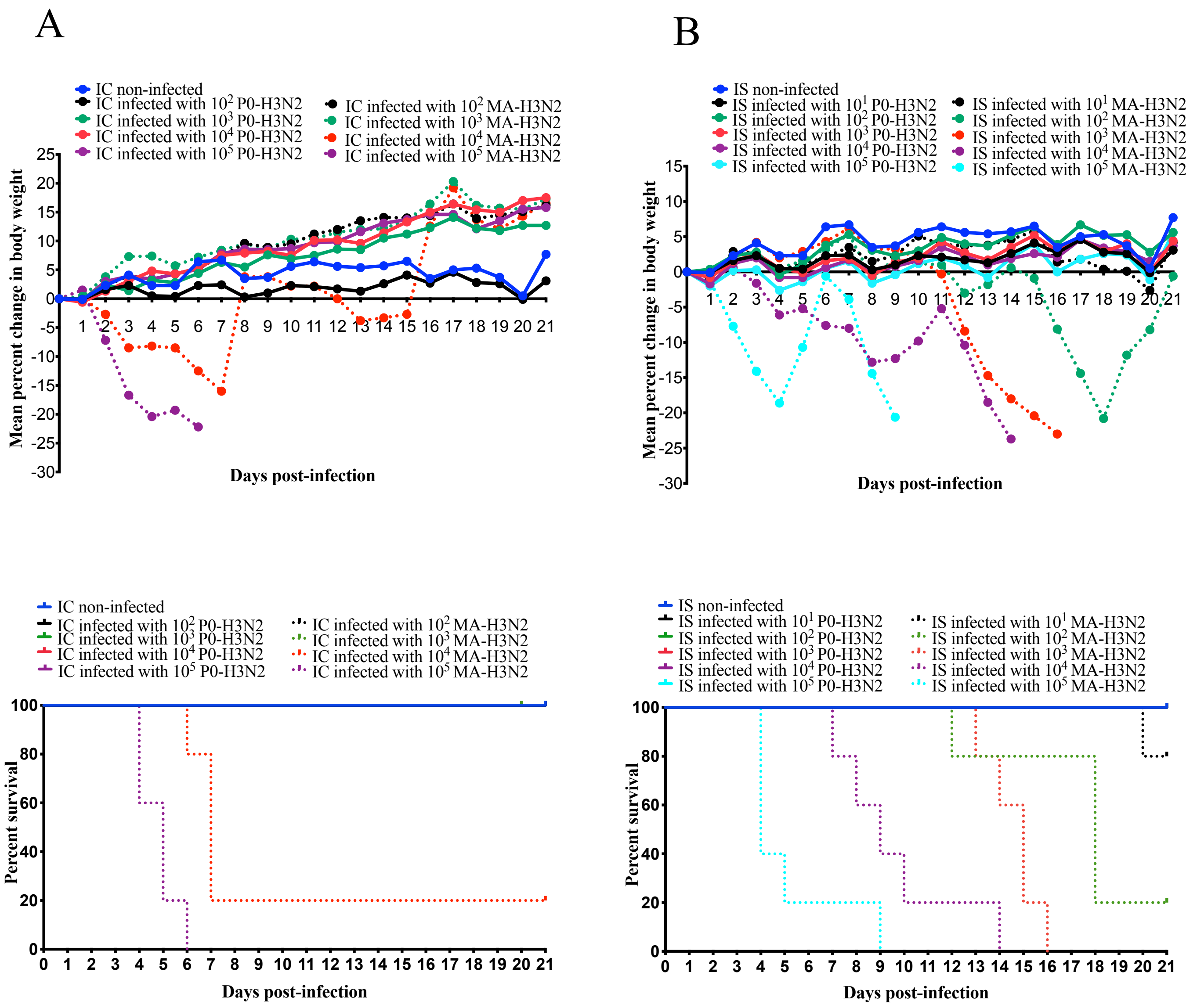

Supplementary figure

Virulence of P0-H3N2 and MA-H3N2 viruses in IC and IS mice. **(A)** Body weight loss and survival curve of IC mice inoculated with 10^2 to 10^5 PFU/50 µl of P0-H3N2 or MA-H3N2 viruses. **(B)** Body weight loss and survival curve of IS mice inoculated with 10^1 to 10^5 PFU/50 µl of P0-H3N2 or MA-H3N2 viruses. Mice were observed daily for 21 days for clinical signs of illness, including weight loss, ruffled fur, and hunching and were sacrificed if they lost ≥20% of their original body weight.
